# The lemon genome and DNA methylome unveil epigenetic regulation of citric acid biosynthesis during fruit development

**DOI:** 10.1093/hr/uhae005

**Published:** 2024-01-05

**Authors:** Hang Yu, Chao Zhang, Chuang Lu, Yana Wang, Congcong Ge, Guixiang Huang, Haifeng Wang

**Affiliations:** State Key Laboratory for Conservation and Utilization of Subtropical Agro-Bioresources, Guangxi Key Lab of Sugarcane Biology, College of Agriculture, Guangxi University, Nanning, Guangxi 530004, China; State Key Laboratory for Conservation and Utilization of Subtropical Agro-Bioresources, Guangxi Key Lab of Sugarcane Biology, College of Agriculture, Guangxi University, Nanning, Guangxi 530004, China; State Key Laboratory for Conservation and Utilization of Subtropical Agro-Bioresources, Guangxi Key Lab of Sugarcane Biology, College of Agriculture, Guangxi University, Nanning, Guangxi 530004, China; State Key Laboratory for Conservation and Utilization of Subtropical Agro-Bioresources, Guangxi Key Lab of Sugarcane Biology, College of Agriculture, Guangxi University, Nanning, Guangxi 530004, China; State Key Laboratory for Conservation and Utilization of Subtropical Agro-Bioresources, Guangxi Key Lab of Sugarcane Biology, College of Agriculture, Guangxi University, Nanning, Guangxi 530004, China; State Key Laboratory for Conservation and Utilization of Subtropical Agro-Bioresources, Guangxi Key Lab of Sugarcane Biology, College of Agriculture, Guangxi University, Nanning, Guangxi 530004, China; Key Laboratory of Crop Cultivation and Physiology, Education Department of Guangxi Zhuang Autonomous Region, Guangxi University, Nanning 530004, China; State Key Laboratory for Conservation and Utilization of Subtropical Agro-Bioresources, Guangxi Key Lab of Sugarcane Biology, College of Agriculture, Guangxi University, Nanning, Guangxi 530004, China; Key Laboratory of Crop Cultivation and Physiology, Education Department of Guangxi Zhuang Autonomous Region, Guangxi University, Nanning 530004, China

## Abstract

Citric acid gives lemons their unique flavor, which impacts their sensory traits and market value. However, the intricate process of citric acid accumulation during lemon fruit growth remains incompletely understood. Here, we achieved a chromosomal-level genome assembly for the ‘Xiangshui’ lemon variety, spanning 364.85 Mb across nine chromosomes. This assembly revealed 27 945 genes and 51.37% repetitive sequences, tracing the divergence from citron 2.85 million years ago. DNA methylome analysis of lemon fruits across different developmental stages revealed significant variations in DNA methylation. We observed decreased CG and CHG methylation but increased CHH methylation. Notably, the expression of RdDM pathway-related genes increased with fruit development, suggesting a connection with elevated CHH methylation, which is potentially influenced by the canonical RdDM pathway. Furthermore, we observed that elevated CHH DNA methylation within promoters significantly influenced the expression of key genes, critically contributing to vital biological processes, such as citric acid accumulation. In particular, the pivotal gene *phosphoenolpyruvate carboxykinase* (*ClPEPCK*), which regulates the tricarboxylic acid cycle, was strikingly upregulated during fruit development, concomitant with increased CHH methylation in its promoter region. Other essential genes associated with citric acid accumulation, such as the MYB transcription factor (*ClPH1/4/5*) and *ANTHOCYANIN 1* (*ClAN1*), were strongly correlated with DNA methylation levels. These results strongly indicate that DNA methylation crucially orchestrates the metabolic synthesis of citric acid. In conclusion, our study revealed dynamic changes in DNA methylation during lemon fruit development, underscoring the significant role of DNA methylation in controlling the citric acid metabolic pathway.

## Introduction

In eukaryotes, DNA methylation is a conserved and reversible epigenetic marker that plays important roles in maintaining genome stability, mediating transposon silencing, and regulating gene expression [1–4]. DNA methylation plays important regulatory roles in biological processes in plants, such as floral organ development, seed germination, fruit ripening, and responses to environmental stresses [5–8]. Unlike the sequence context of DNA methylation in mammals, DNA methylation in plants consists of three different sequence forms: two symmetric sequence forms of DNA methylation, CG and CHG (H for A, T, or C), and an asymmetric sequence form, CHH. In plants, DNA methylation involves two processes: *de novo* establishment and maintenance. The *de novo* establishment of DNA methylation in plants relies on the RdDM pathway. After the *de novo* establishment of DNA methylation, the maintenance mechanism of DNA methylation occurs along with DNA replication, which is responsible for maintaining the stability of DNA methylation during cell division, growth, and development. In the model plant *Arabidopsis thaliana,* MET1 (METHYLTRANSFERASE 1) and CMT3 (CHROMOMETHYLTRANSFERASE 3) maintain the symmetric sequence forms of CG and CHG site methylation, respectively [9], whereas the asymmetric sequence form of CHH site methylation is produced via the CMT2 and RdDM pathways [10]. DNA methylation studies can provide novel research perspectives and theoretical support for understanding plant evolutionary relationships and for breeding for superior traits.

**Figure 1:**
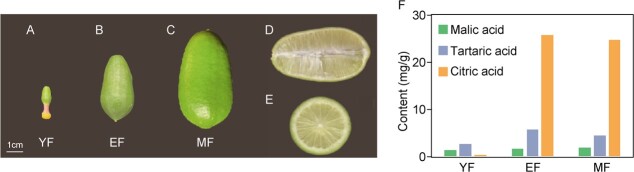
Features of lemon. (**A**) Young fruit (YF). (**B**) Expanding fruit (EF). (**C**) Mature fruit (MF). (**D**) Vertical cross-section of a lemon. (**E**) Cross-sectional view of a lemon. (**F**) Barplot showing the content of three representative organic acids across three developmental stages of lemons.

Lemons are among the most distinguished plants of the *Citrus* genus. Among them, ‘Xiangshui’ lemons [*Citrus limon* (L.) Burm f.] have emerged as a remarkable cultivar from Taiwan, China, boasting a plethora of outstanding attributes [11]. These attributes include its unique ability to flower and bear fruit throughout the year, absence of seeds, early fruit maturation, and a commendably high yield. Its distinct features of oblong and yellowish-green or ripe yellow fruits, smooth exteriors, and robust flavor contribute to its unmistakable identity (Fig. 1A–E; Fig. S1, see online supplementary material). Beyond their visual appeal, lemons hold a place of considerable health significance, containing essential vitamins, citric acid, malic acid, and various other beneficial compounds. Citric acid, a pivotal component of lemons, is the most abundant organic acid (Fig. 1F) [12, 13]. Its synthesis and accumulation form a pivotal biochemical process within plants, with considerable influence over diverse domains, such as growth, development, metabolic orchestration, and fruit quality. This complex process is affected by both substrate supply and regulation by multiple enzymes and gene expression. Sugar metabolism and the pyruvate pathway were prerequisites for citric acid synthesis. The tricarboxylic acid cycle (TAC) and several other regulatory pathways produce citric acid and affect its accumulation. The synthesis and accumulation of citric acid are complex processes influenced by multiple biochemical pathways and regulatory mechanisms [14, 15]. Currently, most studies on citric acid in lemons are at the level of component determination, market value, and anti-disease applications, with less research on the molecular mechanisms, especially epigenetic-related studies, which are urgently needed.

DNA methylation has emerged as a pivotal player in shaping various aspects of *Citrus* fruit growth and development, including phenotypic diversity and responses to ever-changing environments. This epigenetic modification has been particularly elucidated in sweet oranges [16], where a sweeping increase in DNA methylation during fruit ripening exerts a profound impact on vital processes such as photosynthesis. It also influences the expression of pivotal enzymes including abscisic acid. Furthermore, in blood oranges subjected to cold treatments, alterations in DNA methylation patterns intricately modulate the expression of genes responsible for anthocyanin synthesis [17], revealing a previously untapped connection between epigenetic regulation and pigmentation in *Citrus* plants. In sour pomelos, DNA methylation changes in key gene promoters are intricately linked to citric acid synthesis. This interplay directly influences the citric acid content within the fruit’s succulent pulp, underlining the far-reaching implications of epigenetic modifications on fruit quality [18]. With these groundbreaking insights, the urgent task is to unravel the complex DNA methylation mechanisms governing essential genes in the citric acid synthesis and accumulation pathways in lemons.

This study aimed to confirm the role of DNA methylation in the regulation of important functional traits during lemon fruit development. Therefore, we carried out whole-genome sequence sequencing and assembly of ‘Xiangshui’ lemons using PacBio, Illumina, and Hi-C sequencing technologies and analysed the evolutionary relationship of the genome of lemons with that of other *Citrus* plants as well as the genetic variation of the genome through comparative genomics. After assembling the lemons reference genome, methylation and transcriptome sequencing of multiple lemon tissues (young, expanding, and mature fruits) were performed using high-throughput sequencing technology. We comprehensively applied genomics, epigenomics, and transcriptomics to explore the changing patterns of DNA methylation during different developmental periods of lemon fruits and the relationship between genes important for citric acid synthesis and accumulation and DNA methylation. Our study will not only help to reveal the evolutionary relationship of lemon species but also enrich new knowledge of the developmental mechanism of DNA methylation regulation in fleshy fruits and provide a theoretical basis and technical support for breeding high-quality lemon varieties in terms of genetics and epigenetics.

## Results

### Lemon genome sequencing, assembly, and annotation

**Figure 2:**
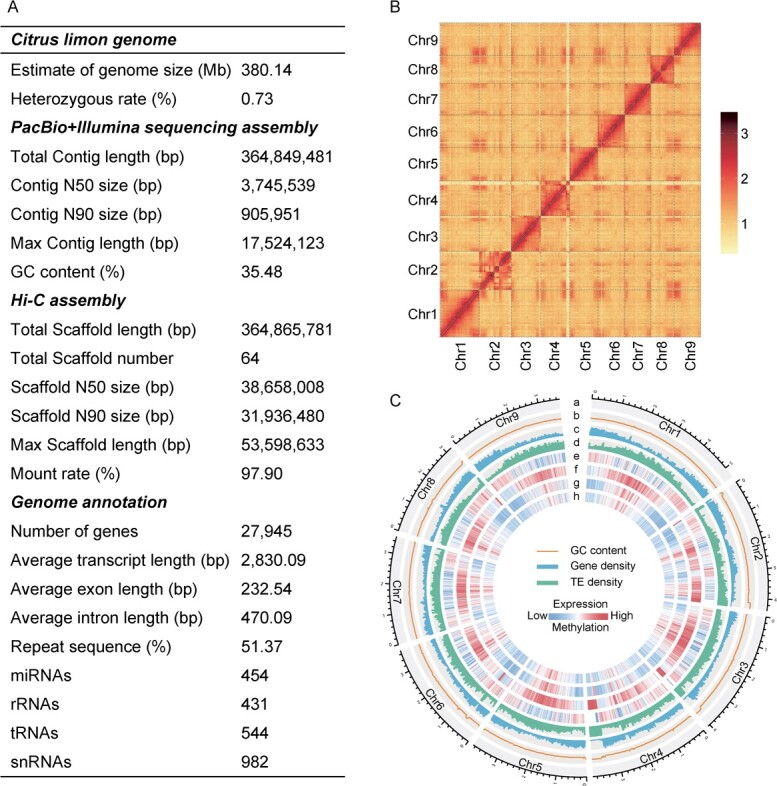
Genomic characteristics of the *Citrus limon* assembly and annotation. (**A**) The table showing essential information about the genome assembly and annotation of *C. limon.* (**B**) Hi-C contact map of the chromosome-scale assembly of *C. limon*. (**C**) Circos plot showing: (a) chromosomes of *C. limon*; (b) GC content; (c) gene density; (d) TE density; (e) gene expression level; (f) CG methylation level; (g) CHG methylation level; (h) CHH methylation level (bin size = 200 kb).

We selected the ‘Xiangshui’ lemon variety from Guangxi, China for genome sequencing. Short-reads sequencing using the Illumina platform generated 53.2 Gb of data (~139.95 X depth). Genome survey analysis (K = 17) predicted a size of 380.14 Mb with 0.73% heterozygosity and 57.9% repetitive sequences (Fig. 2A; Fig. S2, see online supplementary material). To achieve a high-quality genome, we generated 50.23 Gb (395.20 X depth) of PacBio reads for the initial assembly and used Illumina short reads (53.3 Gb) to correct errors. Consequently, we achieved a contig level assembly of 364.85 Mb with an N50 of 3.75 Mb and GC content of 35.48% (Fig. 2A; Table S1, see online supplementary material). The assembly was elevated to the chromosome level using Hi-C sequencing (48.64 Gb, ~127.95 X depth), resulting in nine pseudo-chromosomes (Fig. 2B; Table S2, see online supplementary material), with an anchor rate of 97.9%. Genomic integrity assessment metrics showed that Benchmarking Universal Single-Copy Orthologs (BUSCO) reached 95% (Table S3, see online supplementary material), the Core Eukaryotic Genes Mapping Approach (CEGMA) reached 97.18% (Table S4, see online supplementary material), and BWA compared the second-generation data to the genome with a 99.06% alignment ratio, covering 99.96% of the genome (Table S5, see online supplementary material), indicating a high-quality lemon reference genome.

A comprehensive strategy encompassing *de novo* prediction, homology prediction, and transcriptome-based analysis was employed to annotate the protein-coding genes, repeat sequences, and non-coding RNAs. Annotation results revealed 27 945 coding genes in the lemon genome. The average transcript length was 2830.09 bp, whereas the coding sequence length reached 1092.20 bp, with an average of 4.70 exons per gene. Notably, 98.5% of the genes exhibited predictable function when their predicted protein sequences were matched with known protein libraries (Fig. 2C).

**Figure 3:**
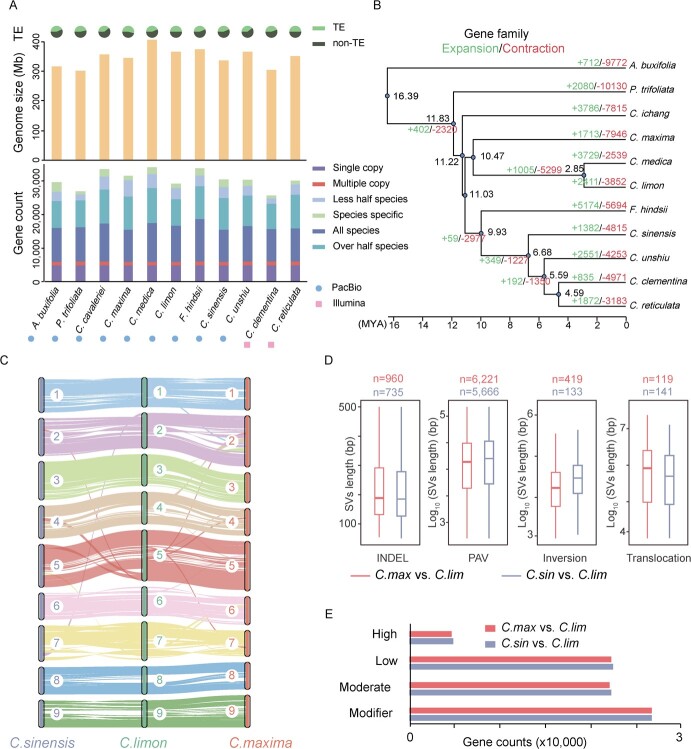
Comparative genomic analysis of *Citrus limon*. (**A**) From top to bottom: TE proportion, genome size, and gene counts across different species. (**B**) Expansion and contraction of gene families among 11 species. Phylogenetic analysis was performed based on the single-copy genes among 11 species. (**C**) Genome collinearity among *Citrus sinensis (C. sin)*, *Citrus limon (C. lim),* and *Citrus maxima (C. max)*. (**D**) Box plots showing the length distribution of different categories of structural variants in *C. max* vs. *C. lim* and *C. sin* vs. *C. lim*, respectively. (**E**) Barplots showing the number of genes affected by different categories of structural variants in *C. max* vs. *C. lim* and *C. sin* vs. *C. lim*, respectively.

Repeat sequences constituted 51.37% of the genome, with transposable elements (TEs) accounting for 48.30%. Most of the repeat sequences were long terminal repeat (LTR) retrotransposons, among which copia-type LTR accounted for 13.40% and gypsy-type LTR accounted for 17.79%. Long interspersed nuclear element (LINE) retrotransposons and DNA types accounted for 2.06% and 4.55% of the genome, respectively (Table S6, see online supplementary material). A total of 454 miRNAs, 544 tRNAs, 431 rRNAs, and 982 snRNAs were identified in the lemon genome. Collectively, we performed high-quality assembly and annotation of the lemon genome for subsequent analyses.

### Comparative analysis of lemon and *Citrus* species

The *Citrus* genus displays a distinct profile marked by frequent hybridization, a plethora of species, and an intricate evolutionary history [19, 20]. Comparative genomic analysis has revealed rapid evolutionary dynamics in plants, thereby shedding light on species-specific adaptations during the evolutionary process. To gain a deeper understanding of the evolutionary process of lemons, we selected *Poncirus trifoliata* (a.k.a. *Citrus trifoliata*)*, Citrus cavaleriei*, *Citrus medica, Citrus grandis* (a.k.a. *maxima*), *Fortunella hindsii* (a.k.a. *Citrus hindsii*), *Citrus sinensis, Citrus unshiu, Citrus reticulata, Citrus clementina,* and *Atalantia buxifolia* as an outgroup of the *Rutaceae* family [21]. These ten species were analysed for genomic comparisons with lemons. We found that the genome sizes of *Citrus* spp. were concentrated in the range of 303–406 Mb, with the largest being *C. medica* and the smallest *P. trifoliata*. This size is consistent with the genome sizes of other species in the Rutaceae family, such as *Murraya koenigii* (331.7 Mb) and *Luvunga scandens* (342.8 Mb). One exception is *Zanthoxylum bungeanum*, which has a genome size of up to 4 Gb. The number of protein-coding genes in the 11 species ranged from 24 533 to 32 257, and showed a significant positive correlation with genome size (R = 0.82, *P* = 0.002), whereas the content of TEs did not show this correlation (Fig. 3A). A total of 320 591 genes in these 11 species were identified in 29 188 gene families, of which 77 families were unique to lemons. These unique families were mainly involved in the cellular response to far-red light, light stimulus, and radiation, positive regulation of gene expression, and epigenetic and other important biological functions (Table S7, see online supplementary material). Meanwhile, we identified 4778 single-copy gene families in 11 species and constructed a phylogenetic tree (Fig. 3B). We found that lemons and *C. medica* diverged closest to each other, with a divergence time of approximately 2.85 million years ago (MYA), and *A. buxifolia* diverged approximately 16.39 MYA. The topology of the tree was consistent with previous studies [18, 22]. To explore the evolutionary history of gene families in citrus species, we analysed the expansion and contraction of gene families using CAFE, and found 2411 expanding families and 3852 contracting gene families in lemons compared to other species. We found that the main functions of these gene families are involved in flavonoid metabolism, pigment metabolic processes, regulation of RNA splicing, vacuolar acidification, the protein ADP-ribosylation, pH reduction, and other important biological processes (Fig. S3, see online supplementary material).

Structural variation (SV) is a crucial mechanism driving genetic evolution in plant genomes. Within the *Citrus* genus, it serves as the primary cause of phenotypic differences between species [23, 24]. In this study, we focused on pomelos (*C. max*), which are evolutionarily closer to lemons, and the extensively researched sweet oranges (*C. sin*), both of which possess more complete genomes. Our analysis examined the structural variability between these species and lemons. Initially, we compared the genomes of lemons with those of pomelos and sweet oranges, revealing significant collinearity among their genomes. However, numerous distinct types of SVs were observed (Fig. 3C). By comparing each of these species to lemons, we identified specific structural variations between lemons and pomelos (*C. max* vs. *C. lim*), as well as between lemons and sweet oranges (*C. sin* vs. *C. lim*). The comparison yielded a total of 3 239 195 and 4 607 976 single-nucleotide polymorphisms (SNPs) between lemons and pomelos, and between lemons and sweet oranges, respectively. The number and length distributions of other types of SVs were depicted in Fig. 3D. SV impacts species differentiation and can affect the genome to varying degrees depending on where the variation occurs [25]. Based on the degree of effect of SVs on genes, we classified SVs into four categories: high, moderate, low, and modifier. Among these four categories of SVs, modifier SVs have the least significant effect on genes compared to the other three. Additionally, the number of genes associated with this category of SVs is the highest. In contrast, the lowest number of genes was associated with high-impact SVs. Notably, we found that the number of genes affected by the four categories of SVs was similar in lemons compared to sweet oranges and pomelos, respectively (Fig. 3E).

In conclusion, we determined the genomic and gene family profiles of several species of the genus *Citrus* through comparative genomics approaches, identified evolutionary information and endemic families in lemons, and identified key genes in lemons that are strongly affected by SVs based on the analysis of SVs in pomelos and sweet oranges.

### Characterization of DNA methylome during lemon fruit development

**Figure 4:**
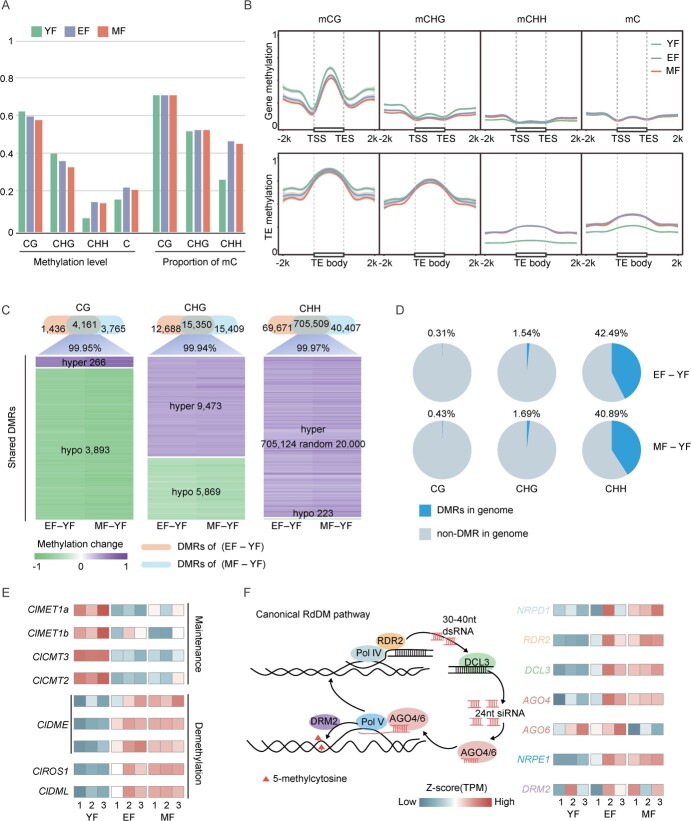
DNA methylation variations across different development stages of lemon fruit. (**A**) Barplot showing genome-wide methylation levels and the proportion of methylcytosine for the three periods in the CG, CHG, and CHH contexts. (**B**) The metaplot showing DNA methylation patterns and levels of protein-coding genes and transposons of three different stages. (**C**) Comparisons of DMRs between EF-YF and MF-YF. Venn diagram showing comparison of the number of DMRs in CG, CHG, and CHH context. The heatmap showing the methylation level change of DMRs shared by EF-YF and MF-YF groups. (**D**) The pie chart showing the proportion of DMRs across the genome. (**E**) The heatmap showing the expression changes of representative genes encoding DNA methylase and demethylase of three fruit development stages. (**F**) Schematic outlining the model and expression changes of pivotal genes involved in the RdDM pathway.

To explore the dynamic changes in DNA methylation during lemon fruit development, we selected small lemon fruits (young fruit; YF), expanding fruits (expanding fruit; EF), and mature fruits (mature fruit; MF) (Fig. 4A) to generate DNA methylation profiles with single-base resolution via whole genome bisulfite sequencing (WGBS). More than 99% of the cytosines were successfully transformed by the bisulfite treatment. For each biological repeat, at least 99 508 398 reads were generated, covering more than 40X of the genome, and at least 70.79% of the reads were uniquely aligned to the reference genome, covering at least 97.19% of the cytosines in the genome (Table S8, see online supplementary material). Correlation analysis (Fig. S4, see online supplementary material) and principal component analysis (PCA) (Fig. S5, see online supplementary material) demonstrated high consistency among replicates. In conclusion, the quality and depth of our sequencing data were excellent compared to other experiments [26, 27] and can be used for subsequent studies.

First, we calculated the genome-wide methylation levels of lemon fruits in different contexts. It was found that the methylation changes in CG and CHG were consistent with each other, with methylation levels gradually decreasing during lemon development. However, CHH was different, experiencing an increasing and then a slightly decreasing trend (0.066 in YF, 0.153 in EF, and 0.145 in MF, Fig. 4A). We found that genome-wide cytosine methylation levels followed the same trend as CHH, suggesting that changes in CHH methylation levels are the main factors influencing genome-wide methylation levels during lemon fruit development. Meanwhile, the proportions of methylcytosines occurring at different periods in different contexts were calculated using a binomial test (*P*-value <0.05). We observed no significant changes in the methylcytosine proportion in the CG and CHG contexts, whereas the trend of the methylcytosine proportion in the CHH context was consistent with the methylation levels (27.26% for YF, 46.83% for EF, and 46.03% for MF) (Fig. 4A). These results suggest that the changes in methylation levels in CG and CHG may occur in the same methylcytosines, whereas the elevated methylation levels in CHH were due to the creation of many new methylcytosine sites (Fig. S6, see online supplementary material).

After exploring the overall methylation levels of the genome during lemon development, we examined the methylation levels and patterns in the genetic and TE regions of the genome. These results were consistent with the whole-genome methylation trend, with CG and CHG experiencing a gradual decline, and an increase followed by a slight decrease from YF to EF to MF in the CHH context. It was also found that the methylation level of TE was high in the body region, whereas in the body (TSS-TES) region of protein-coding genes, the methylation level of CG was significantly higher than that of the non-CG contexts (Fig. 4B; Fig. S7, see online supplementary material), which is consistent with the methylation trend in other *Citrus* genera as well as in a variety of plants [28, 29].

Next, we identified differentially methylated regions (DMRs) in the lemon genome to gain further insights into changes in methylation. The genome was divided into 200 bp windows and methylation differences were calculated for each window in the EF-YF and MF-YF groups, defining windows CG > 0.4, CHG > 0.2, and CHH > 0.1 (*P*-value <0.05 by Fisher’s exact test and q-value <0.05 by FDR) as DMR. We observed that CHH possessed the largest proportion of shared DMRs and almost all shared DMRs were in the same orientation (99.95% in CG, 99.94% in CHG, and 99.97% in CHH) (Fig. 4C), suggesting that most changes in DNA methylation during lemon development, especially in the CHH context, occurred in the same region. DMRs in the CHH context influenced the genome to a greater extent than in the CG and CHG contexts, with more than 40% of the genome receiving CHH differential methylation (Fig. 4D). In summary, we identified methylation variants that change during lemon development and found that CHH not only has a greater effect on genome-wide methylation levels but also has the widest range of effects across the genome.

### Increased expression of RdDM-related genes during lemon fruit development

Cytosine methyltransferases and demethylases in plants play important roles in the maintenance of methylation and de-methylation, both of which are involved in various biological processes [30, 31]. Methyltransferases and demethylases in the lemon genome were obtained by sequence alignment with genes previously identified in *A. thaliana* and mapped to genome annotation files [10, 32]. We observed that *ClMET1* (for CG) and *ClCMT3* (for CHG), which are responsible for maintaining methylation, showed higher expression in YF than in EF and MF stages, which is consistent with the methylation levels of CG and CHG. The demethylases *ClDME*, *ClROS1*, and *ClDML* showed opposite expression trends compared to the methyltransferases (Fig. 4E), which was consistent with the methylation variation, suggesting that the methylation levels of CG and CHG are jointly regulated by methyltransferases and demethylases.

For CHH methylation, the trend of methylation variation was not consistent with the expression levels of methyltransferases and demethylases, which was due to the specific structure of the CHH context, which was mainly established from *de novo* through the RdDM pathway. We used the same method to identify the key genes involved in the RdDM pathway in lemon and analysed their expression patterns. With the exception of *ClAGO6*, gene expression in the RdDM pathway was lower in YF than in EF and MF, which was consistent with the genome-wide methylation pattern of CHH. Therefore, it was inferred that methylation changes in CHH during lemon development were mainly regulated by the RdDM pathway (Fig. 4F).

### Hyper-CHH DNA methylation effects gene expression

**Figure 5:**
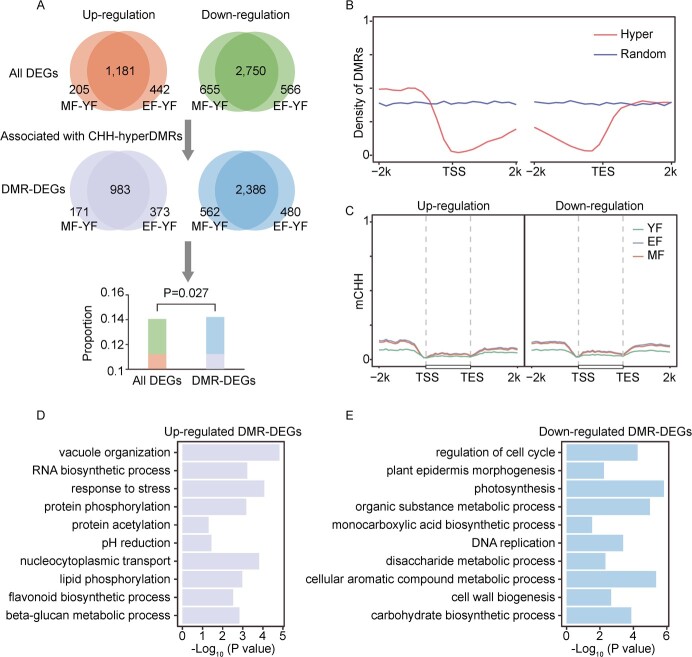
Association between hyper-DMR of CHH context and differential expression genes. (**A**) Comparisons of DEGs and DMRs of MF-YF and EF-YF groups, respectively. The Venn diagram demonstrates the number of overlapping DEGs and DMR-associated DEGs of MF-YF and EF-YF groups. The barplot showing the statistics of DEGs and DMR-associated DEGs. *P*-values were determined using the hypergeometric test. (**B**) Comparative analysis of the distribution of DEGs associated with CHH hyper-DMRs with randomly selected genomic regions. (**C**) The metaplot showing the CHH methylation level and pattern of DEGs associated with CHH-hyper DMRs. (**D**–**E**) The barplot showing GO enrichment analysis of up-regulated (**D**) and down-regulated (**E**) DEGs associated with CHH-hyper DMRs.

To investigate the relationship between DNA methylation and gene expression during lemon development, transcriptome sequencing was performed on lemon pulp from the YF, EF, and MF periods, with three biological replicates from each sample. Each replicate sequencing generated at least 38 939 092 reads, of which at least 85.15% uniquely matched to the reference genome (Table S9, see online supplementary material). Correlation analysis (Fig. S8A, see online supplementary material) and PCA (Fig. S8B, see online supplementary material) between replicates showed a very high similarity and the sequencing quality was sufficiently high to proceed to the next step of the analysis. Similar to methylation analysis, we calculated the differentially expressed genes (DEGs) in MF-YF and EF-YF groups. There were 1828 and 3971 DEGs upregulated and down-regulated in EF and MF, respectively, compared to YF (Log_2_|FC| > 2, adjusted *P*-value <0.05). Among them, 64.61% (1181) and 69.25% (2750) were common to EF and MF, respectively. However, only 229 and 124 DEGs were upregulated and downregulated in the EF to MF period. This suggested that the genome underwent significant expression changes from YF to EF, followed by smaller gene expression changes from EF to MF, resulting in a stable overall gene expression profile (Fig. 5A).

Previous studies have indicated that genomic methylation changes were mainly caused by elevated methylation in the CHH context. To analyse the effect of DNA methylation on gene expression in the context of CHH, we defined differential methylation genes (DMG) as a gene and its upstream and downstream 2 kb region with intersecting DMRs. We found that 85.4% (4955 in DMGs/5799 in all genes) of the DEGs were DMGs, and the proportion of DEGs in DMGs was significantly higher than that in all genes (*P*-value = 0.027, hypergeometric test, Fig. 5A). This suggests that genes with methylation changes are more likely to exhibit differential expression, indicating that DNA methylation plays a role in differential gene expression during lemon development.

To analyse the specific mechanism of this effect, we assessed the distribution of DMRs among DEGs. We found that the densities of CHH-hyper DMRs in the promoter and downstream regions of TES were significantly higher than the random densities of the genomes, especially upstream of the TSS (Fig. 5B). This phenomenon directly led to significant changes in the CHH methylation of these DEGs upstream of the TSS and downstream of the TES (Fig. 5C). This suggests that DMRs located upstream and downstream of genes, particularly in the promoter region, may regulate gene expression. Meanwhile, we performed GO functional enrichment analysis on the upregulation and downregulation of these DEGs affected by DMRs and found that these genes were mainly involved in processes related to important phenotypes of lemons, such as vesicle organization, pH lowering, protein phosphorylation, and monocarboxylic acid biosynthesis, lipid phosphorylation, disaccharide metabolism process, photosynthesis, and other biological processes related to plant growth and development (Fig. 5D and E).

### DNA methylation regulates genes involved in citric acid biosynthesis

**Figure 6:**
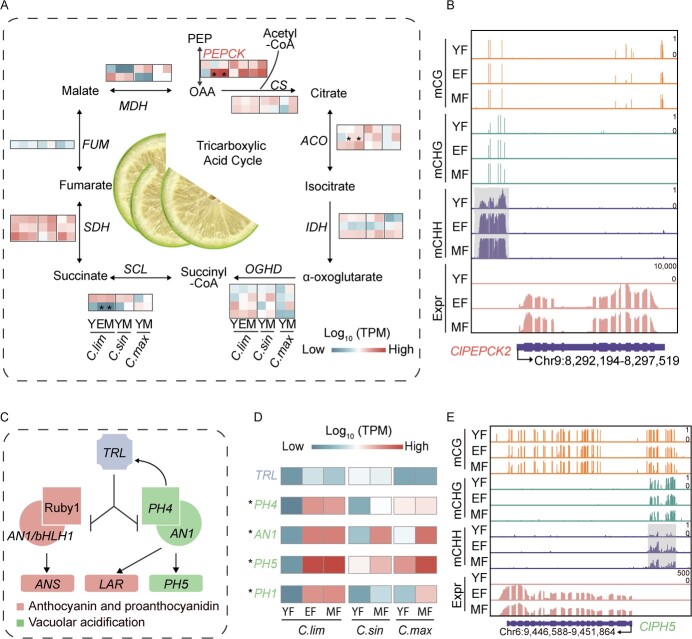
Increased CHH methylation contributes to citric acid biosynthesis of lemon fruit. (**A**) Schematic diagram depicting the tricarboxylic acid cycle in lemons. Heatmaps display gene expression levels in lemons (*C. lim*), sweet oranges (*C. sin*), and pomelos (*C. max*) at various developmental stages. The asterisk (*) signifies a differentially expressed gene (DEG) in lemon fruit. (**B**) The genome browser showing the DNA methylation level in the CHH context and the expression level of *ClPEPCK2*. (**C**) Recent proposed fruit acidification model of *Citrus* species [34]. Working model delineating the role of TRL in cross-regulating vacuolar acidification and the biosynthesis of anthocyanin and proanthocyanidin. (**D**) The heatmap showing the expression changes of genes involved in (**C**) model. The asterisk (*) indicates that the gene is the DEG in lemon development. (**E**) Genome browser showing the CHH methylation variation in the CHH context and the expression level of *ClPH5*. Y/YF stands for young fruit. E/EF stands for expanding fruit. M/MF stands for mature fruit.

We found significant changes in DNA methylation during lemon fruit development, especially CHH methylation, and these methylation changes were associated with differential expression of key genes for fruit acidity and the synthesis of important acids. To explore the major components of lemon fruit acidy, we analysed the organic acid content of fruit at three different developmental stages. Malic acid was found to remain relatively stable, tartaric acid increased slightly with development. The content of citric acid increased significantly from YF to EF stage (Fig. 1F), which was consistent with the trend of methylation levels (Fig. 4A). This suggests that citric acid may be an important component of lemon fruit acidity and its biosynthesis may be related to DNA methylation. We know that citric acid is an important metabolite of the tricarboxylic acid cycle (TAC) [33]. Therefore, we investigated the expression patterns of essential genes involved in TAC to gain insight into citric acid accumulation. We found that the expression of many genes was stable across different stages, except for *PEPCK*, *ACO*, and *SCL*. In addition, we downloaded transcriptome data from public databases for different fruit development stages of sweet oranges and pomelos (YF and MF stages). Similarly, there were no significant changes in the expression of any of the genes except for *PEPCK*, which was significantly increased from YF to EF. In contrast, the expression of PEPCK was higher in lemon during the ripening stage than in sweet oranges and pomelos (Fig. 6A), and we believe that this can partially reveal that citric acid content in lemon fruits is higher than in sweet oranges and pomelos. PEPCK is the key enzyme responsible for the synthesis of OAA, which is the first step in the synthesis of citric acid from acetyl coenzyme A in the presence of citrate synthase (CS) [34, 35]. Notably, we found a significantly higher level of CHH methylation of the *ClPEPCK* promoter region from YF to EF stage. This elevated methylation level appeared to promote *ClPEPCK* expression (Fig. 6B). Therefore, we hypothesized that hypermethylation of the *ClPEPCK* promoter region caused an up-regulation of its expression and increased OAA synthesis, which in turn led to an increase in citric acid production. These findings are consistent with previous studies that have shown a role for PEPCK in promoting the accumulation of organic acids in various fruits [36].

The synthesis and accumulation of citric acid in plants are complex processes. In addition to the well-known TAC, a biological process known as the TRIPTYCHON-LIKE (TRL) model exists in *Citrus* plants that affects citric acid content [34] (Fig. 6C). In this model, TRL is a negative regulator that is activated by PH4 (encoding an MYB transcription factor), which then competes with PH4 for the binding of AN1. The binding of PH4 and AN1 promotes the expression of *PH5*, which in turn promotes the synthesis and accumulation of citric acid. The positive regulation of citric acid content by *PH4, AN1, PH5*, and *PH1* has been validated in many studies [18, 37–39]. We then analysed the genes associated with citric acid synthesis in this model and compared their expression changes in lemon with those of sweet oranges and pomelos during different stages of fruit development. Expectedly, we observed that all genes except TRL, which had a negative regulatory role, underwent significant increase in the expression of all genes in lemon from YF to EF stage, such as *PH4*, *AN1*, *PH5*, and *PH1*. Notably, these genes had higher expression at later stages in lemons compared to sweet oranges and pomelos (Fig. 6D), which suggests that TRL has no inhibitory effect on the citric acid accumulation, whereas Ph4, AN1, PH5, and PH1 promoted citric acid accumulation. It is noteworthy that all of these genes had numerous of CHH-DMRs in their promoter regions (Fig. 6E; Fig. S9, see online supplementary material). This suggests that DNA methylation promotes citric acid accumulation during lemon development by regulating the expression of key genes in the TRL model.

## Discussion

In this study, we focused on the ‘Xiangshui’ lemon variety from the Guangxi region, China and conducted comprehensive genome, transcriptome, and epigenome studies. The ‘Xiangshui’ lemon stands out with unique characteristics, such as multiple flowering and fruiting throughout the year, fragrant and seedless ripe fruits, and noteworthy traits like self-incompatibility. Despite the release of the lemon genome in previous studies [22, 40], it was essential to sequence the genome of excellent varieties like the ‘Xiangshui’ lemon. To achieve this, we adopted a combination of third-generation and second-generation sequencing strategies, along with Hi-C technology, to assemble the genome at the chromosome level. We achieved a high-quality lemon genome sequence map with a size of approximately 364.86 Mb, and nine pseudo-chromosomes accounting for 97.9% of the genome. We successfully annotated 27 945 genes and discovered that the genome size and gene number of lemons were comparable to other *Citrus* species, with repeats constituting 51.37% of the genome. Notably, assessments using BUSCO and CEGMA demonstrated high genomic integrity, reaching 95% and 97.18%, respectively (Tables S3 and S4, see online supplementary material), indicating the successful generation of a high-quality lemon reference genome. This high-quality whole-genome sequence serves as the fundamental basis for various multi-population studies, including genetic analyses, functional genomics, and pan-genomics investigations. Additionally, the genome provides vital insights into evolutionary genomics, facilitating the discovery of genes associated with *Citrus* fruit biology and other crucial nutritional traits. Overall, the findings of this study hold great promise for advancing our understanding of *Citrus* genetics and contributing to future advancements in *Citrus* fruit breeding and agriculture.

*Citrus* is the most widely cultivated fruit crop globally and has immense commercial and scientific value. With the successful acquisition of the ‘Xiangshui’ lemon genome, we conducted a high-quality genomic comparison and evolutionary analysis by integrating data from nine other *Citrus* species and one *Rutaceae* species. Through a comparative analysis of genome size, gene number, repeat sequences, and other factors, we found that the gene number of *Citrus* species was significantly positively correlated with genome size. Furthermore, phylogenetic tree construction confirmed the evolutionary status of lemons and elucidated the timing of their differentiation from other closely related species. In the gene family analysis, we observed that *Citrus* species harbored more shared single-copy genes as well as specific gene families unique to lemons. Additionally, the expansion and contraction of certain gene families are associated with crucial biological functions, such as positive regulation of gene expression, epigenetic vacuolar acidification, and intracellular pH reduction (Fig. S3, see online supplementary material). Furthermore, through the examination of structural variations in sweet oranges and pomelos, we identified important biological processes in lemons affected by SVs. Through comparative genomic and evolutionary analyses, we successfully unveiled the phylogenetic relationships and evolutionary processes within *Citrus* as well as its close and distant relatives. This study provides a solid theoretical foundation for the classification, identification, and breeding evaluation of *Citrus* germplasm resources. The findings of this study have significant implications for understanding and harnessing *Citrus* genetic resources and paving the way for new possibilities in *Citrus* breeding and research advancements.

DNA methylation plays a crucial role in regulating fruit ripening in fleshy fruits and is considered one of the most significant regulators, along with hormones and transcription factors [5, 7, 41, 42]. The patterns of DNA methylation variation and regulatory mechanisms during fruit ripening differ across various species, as evidenced by studies of tomatoes, strawberries, and sweet oranges, among others [5, 28, 43]. In the present study, we generated single-base resolution maps of DNA methylation at different stages of lemon fruit development. The pattern of DNA methylation during lemon fruit development was completely different from that of sweet oranges. Specifically, there was a decrease in non-CHH methylation and an increase in CHH DNA methylation, leading to an overall increase in genome-wide methylation levels (Fig. 4A). Our findings suggest that DNA methylation variations during lemon fruit ripening were dynamically co-regulated by the activities of methyltransferases and DNA demethylases (Fig. 4E). Specifically, the increase in CHH methylation levels should be attributed to increased activity of the RdDM pathway. Several key components of the RdDM pathway were significantly upregulated during lemon fruit ripening (Fig. 4F). Consequently, the intensified RdDM activity led to an elevated number of methylcytosines in the genome, thereby increasing the overall genome methylation level (Fig. S6, see online supplementary material). Overall, our study sheds light on the dynamic regulation of DNA methylation during lemon fruit ripening and provides valuable insights into the epigenetic mechanisms involved in this process.

Citric acid accumulation is a critical agronomic trait for *Citrus* plants, as changes in fruit acidity profoundly affect taste and fruit value. Citric acid constitutes a substantial portion of the organic acids in *Citrus* fruits, accounting for 75–97% [12, 44], making it essential to study its accumulation characteristics. The synthesis of organic acids in *Citrus* fruits occurs primarily through the TAC in both the cytoplasm and mitochondria. We found that the expression level of PEPCK, which is responsible for synthesizing OAA during the TAC, increased with fruit development in lemons, and was much higher than that in sweet oranges and pomelos during the ripening stage. PEPCK plays a pivotal role in replenishing intermediate products lost during the TAC for various reasons, making it crucial for the TAC in C_3_ plants. Moreover, PEPCK also promotes seed germination, malic acid, and citric acid synthesis, exerting a significant influence on plant growth and development [45]. Our study reveals that high expression of *ClPEPCK* may be associated with elevated CHH methylation in the promoter region of the gene (Fig. 6B), highlighting the role of DNA methylation in controlling citric acid synthesis through PEPCK regulation. Citric acid synthesis and accumulation are intricate processes. Recent study has identified hypermethylation of the *CgAN1* promoter in pomelos was associated with a reduced citric acid content in the pulp [18]. In addition, the TRL regulatory mode of AN1 plays a crucial role in the biosynthesis of citric acid and proanthocyanidin in *Citrus* plants [34]. Through the analysis of key genes in the TRL model during lemon fruit development, we observed that *TRL* gene did not exhibit any inhibitory effects during fruit ripening, whereas the expression levels of the *ClAN1* and *ClPH4/5/1* increased with fruit development, and most of them were higher than those of sweet oranges and pomelos. Notably, the elevated expression of these genes may be associated with hypermethylation of CHH context in their promoter regions (Fig. 6E; Fig. S9, see online supplementary material). Taken together, DNA methylation could be involved in citric acid synthesis and accumulation by participating in the regulation of the expression of key genes in TAC and TRL pathways.

## Materials and methods

### Plant materials and genome sequencing

‘Xiangshui’ lemons were selected for de novo genome sequencing. The sampled tree was planted in a net shed at the Agricultural College of Guangxi University, and the level of field management was good. Fresh tender leaves were collected and immediately stored in liquid nitrogen at −80°C. The extraction, qualification, and sequencing of genomic DNA were carried out by using Beijing Novogene Bioinformatics Technology. Regarding the published *Citrus* genome data, a 350 bp small fragment library, 20 K large fragment library, and Hi-C library were constructed, and the libraries were sequenced on the PacBio and Illumina HiSeq sequencing platforms. Following quality control procedures and NT comparisons of the sequencing data, 53.3 Gb of original Illumina HiSeq sequencing data were obtained. The genome size and heterozygosity of the ‘Xiangshui’ lemons were estimated by Kmer-17 analysis.

For methylation and transcriptome material and sequencing, the sampling locations were the same as for the genome sequencing samples, and pulp from lemons at the YF, EF, and MF stages was collected for DNA methylation and RNA sequencing. WGBS libraries were prepared using the TruSeq DNA LT kit (Illumina, San Diego, CA, USA). Two biological WGBS libraries were sequenced using Hiseq X10 sequencer (Illumina, San Diego, CA, USA) as paired-end 150-bp reads.

Total RNA was extracted from the pulps of the lemon cultivar ‘Xiangshui’ at YF, EF, and MF stages using the cetyltrimethylammonium bromide (CTAB) method. Three biological libraries were constructed utilizing the VHTS Universal V6 RNA-seq Library Prep Kit, following the guidelines stipulated by the manufacturer, Illumina. Subsequently, all three libraries underwent sequencing on the Novaseq 6000 platform (Illumina, San Diego, CA, USA). This sequencing process generated paired-end reads of 150 base pairs each, contributing to the comprehensive dataset for analysis.

### Genome assembly and evaluation

In the assembly of the ‘Xiangshui’ lemon genome, the PacBio data obtained by sequencing were first self-corrected, and error-corrected preassembled reads were obtained. Then, the third-generation data obtained after error correction were assembled with the overlap-layout-consensus algorithm using Canu [46] software. The assembly results from the previous step were corrected again with the second-generation data in Pilon [47] software to improve the accuracy of the results, and a high-quality consensus sequence was finally obtained. The total length of the contigs was 364.85 Mb, and the contig N50 length was 3.75 Mb [48].

After the assembly results were completed, the integrity and consistency of the genome sequence were evaluated. For the analysis of sequence integrity, the BUSCO [49] (http://busco.ezlab.org/) and CEGMA [50] (http://korflab.ucdavis.edu/datasets/cegma/) evaluation methods were combined, and compared to the assembled genome with BWA software (http://bio-bwa.sourceforge.net/). The comparison rate of reads, the degree of genome coverage, and the depth distribution were calculated, and the integrity of the assembly and the uniformity of sequencing were evaluated.

### Horizontal chromosome assembly

After obtaining the draft genome, the chromosome-level genome was obtained with the help of Hi-C technology. Through Hi-C biotin labeling and genomic DNA extraction from fresh tender leaves of ‘Xiangshui’ lemon, the DNA was randomly broken up into 350 bp fragments with a Covaris crusher and then recovered. After repair, ligation, and purification, a Hi-C library was constructed and sequenced by using Illumina HiSeqPE150 technology. Finally, 48.64 Gb of effective data were obtained for Hi-C analysis.

The effective high-quality Hi-C sequencing data were compared to the draft genome with BWA software [48], and it was shown that high-quality data were obtained. At the same time, the reads near restriction sites were extracted for auxiliary assembly. The number of interactions between contigs was counted, and the contigs were clustered according to the number of interactions and divided into the specified class numbers according to the number of chromosomes in the species. According to the results of clustering, the intensity of each two contig interactions, and the position of the interaction read alignments, the assembled contig/scaffold sequences were mapped to the chromosome level by using LAchesis software [51], and the genome at the chromosome level was obtained.

### Genome annotation

#### Repetitive sequence annotation

The repetitive sequence library obtained by de novo prediction was integrated with the homologous repetitive sequence database Repbase, and the ‘Xiangshui’ lemon genome was then subjected to repeat annotation with RepeatMasker software [52]. The library predicted by Repeat Modeler, Repeat Scout, PILER, and LTR_FINDER [53] software combined with the Rep Base nucleic acid library was integrated by Uclust software according to the principle of 80-80-80, and transposon elements were obtained by using Repeat Masker software to annotate the genome [54, 55].

#### Gene structure annotation

De novo prediction was performed using Augustus [56], GlimmerHMM [57], SNAP (http://homepage.mac.com/iankorf/), GeneID [58], and GENSCAN [59] for species annotation. The included species were pomelos (*C. grandis*), citrons (*C. medica*), sweet oranges (*C. sinensis*), Hong Kong kumquats (*F. hindsii*), wild mandarins (*C. reticulata*), clementines (*C. clementina*), and *Arabidopsis* (*A. thaliana*).

#### Functional annotation

The gene set annotated according to gene structure was compared with the known protein database by using comparison software, and the functional information of the genes was obtained. The protein databases used in this analysis were SwissProt (http://www.uniprot.org/), Nr (http://www.ncbi.nlm.nih.gov/protein) Pfam (http://pfam.xfam.org/), KEGG (http://www.genome.jp/kegg/), and InterPro (https://www.ebi.ac.uk/interpro/).

#### Non-coding RNA annotation

According to the structural characteristics of tRNA, tRNA scan-SE software was used to identify tRNA sequences (http://lowelab.ucsc.edu/tRNAscan-SE/) in the genome. rRNAs were identified by comparing the rRNA sequences of related species with BLASTN [60]. Using the covariance model of the Rfam family, Rfam’s INFERNAL (http://infernal.janelia.org/) software could be used to predict the miRNA and snRNA sequence information in the genome [61].

### Comparative genomic analysis

Gene family classification we performed using the default parameters of OrthoFinder [62]. Single-copy genes of the 11 species extracted from the gene family analysis were compared using MUSCLE v5.1 [63] before constructing maximum likelihood phylogenetic trees using RAxML-NG v1.1.0 [64]. We used CAFE v4.0 [65] to obtain the number of contractions and expansions of gene families in the phylogenetic trees of the 11 species using the output of OrthoFinder as input. SYRI [66] and SnpEff [25] were used to identify the SVs and the extent of the effect of SVs on genes, separately, with the extent categorized as follows https://pcingola.github.io/SnpEff/se_inputoutput/.

### Whole genome bisulfite sequencing data analysis

Raw bisulfite sequencing data were quality control filtered using Trimmomatic [67] and then aligned to our assembled lemon reference genome using BSMAP [68]. For alignment, we allowed six mismatches per read, and only uniquely aligned reads were used for further analysis. We used the reads aligned to the unmethylated lambda bacteriophage to calculate conversion rates. The methylation level of each cytosine was calculated using the BSMAP downstream script methratio.py, and the identification of methylcytosines was obtained from the transformation rate and binomial distribution. The methylation level of each site or region was calculated using the weighted methylation level (#C/#C + #T) method [69]. The genome was divided into 200 bp windows and DMRs were calculated using the software methylKit [70] of the R package. The software ViewBS [71] was used to calculate and display the methylation patterns of the upstream 2 kb to downstream 2 kb regions of the gene and TE.

### Transcriptome sequencing data analysis

Raw RNA sequencing data were quality control filtered using Trimmomatic and then aligned to our assembled lemon reference genome using HISAT2 [72]. When aligning, only uniquely aligned reads were used for further analysis. StringTie [73] was used to calculate transcripts per kilobase million (TPM) for each gene. Differentially expressed genes were calculated using DESeq2 [74] (Log_2_|FC| > 2 and FDR < 0.05).

### GO enrichment analysis

GOATOOLS [75] and hypergeometric tests were used to conduct gene ontology (GO) enrichment analysis for SVs-associated genes and DMRs-associated DEGs. Only GO terms with *P*-values less than 0.05 were used for further analysis.

### Measurement of organic acids

Standard stock solutions of malic acid, tartaric acid, and citric acid were prepared at a concentration of 1.0 mg/ml each. Using these standard stock solutions as the base, a mixed standard solution was prepared with concentration gradients of 5.0, 10.0, 20.0, 30.0, 40.0, and 50.0 mg/L. Sequentially injecting 25 μl of the mixed standard solution into the liquid chromatograph, peak areas were measured. By plotting the concentration of organic acids on the x-axis and the corresponding peak area on the y-axis, standard curves for the three organic acids were generated. Frozen lemon samples (5.0 g) were ground, and a sample extraction solution was prepared. After dilution with ultra-pure water by a factor of 50, the solution was filtered through a 0.22 μm microporous membrane. Measurements conducted on the liquid chromatograph yielded varying peak areas. These values were then substituted into the standard curves, enabling the calculation of the content of various organic acids within the fruit samples.

## Acknowledgements

This work was supported by the Guangxi Natural Science Foundation (No. 2023GXNSFDA026034), National Natural Science Foundation of China (No. 32160142), Sugarcane Research Foundation of Guangxi University (No. 2022GZA002), State Key Laboratory for Conservation and Utilization of Subtropical Agro-bioresources (SKLCUSA-b202302) to H.W., and Science and Technology Major Project of Guangxi (Gui Ke AA22068092) to G.H.

## Author contributions

H.W. and G.H. conceived and designed this project. H.Y. and C.Z. performed genome assembly, annotation, and multi-omics analysis. C.L. and Y.W. planted and collected lemon tissues. C.L., Y.W., and C.G. conducted the experiment. H.Y. and H.W. wrote the manuscript. All authors read and approved the final manuscript.

## Data availability statement

The raw reads generated in this study have been deposited in the NCBI sequence read archive (SRA) with the accession number Sequencing Data PRJNA793193. The genome file has been deposited in the CNCB Genome Warehouse under accession number GWHCBFU00000000. The annotation files for the genomes are stored in the Figshare database at the following link: https://doi.org/10.6084/m9.figshare.22633798.v1. The RNA-seq public data for sweet oranges and pomelos were downloaded from NCBI data with the project numbers PRJNA340305 and PRJNA339650, respectively.

## Conflict of interests 

The authors declare that they have no competing interests in this research.

## Supplementary information

Supplementary data is available at *Horticulture Research* online.

## Supplementary Material

Web_Material_uhae005
