# Supplementary material for: The lemon genome and DNA methylome unveil epigenetic regulation of citric acid biosynthesis during fruit development
Source: Hortic Res. 2024 Jan 5;11(3):uhae005. doi: 10.1093/hr/uhae005 (PMC10923643; doi:10.1093/hr/uhae005)
Supplement: Web_Material_uhae005 [file web_material_uhae005.zip › Supplementary Figures.pdf]

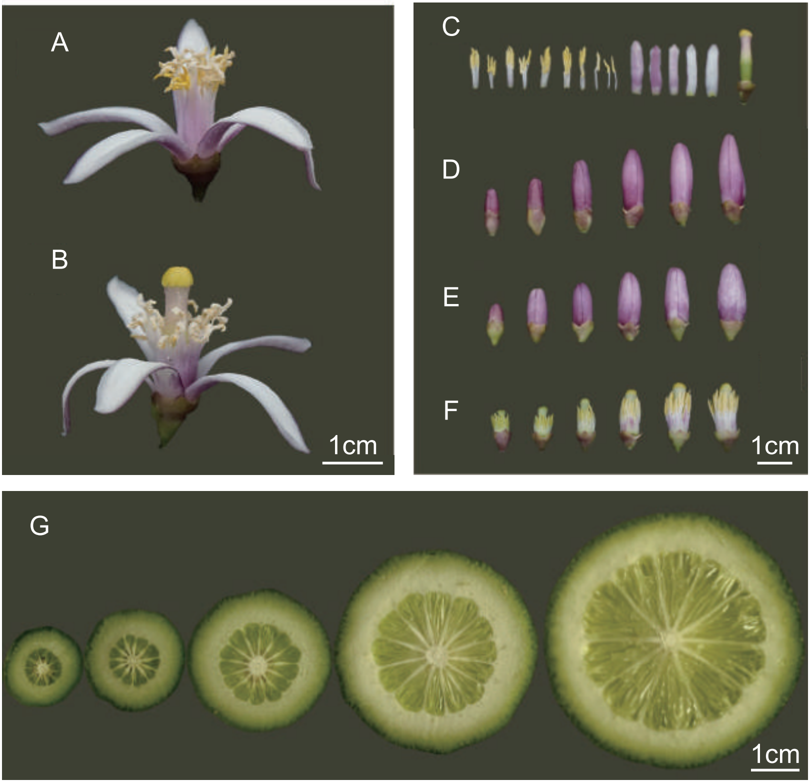

Figure S1. Features of lemon. (A) Male flower. **(B)** hermaphrodite flower. **(C)** The basic structure of the flower. **(D-F)** Growth of male flower buds **(D),** hermaphrodite flower buds **(E),** and pistil and stamen **(F). (G)** Cross-cut of lemons at different development stages.

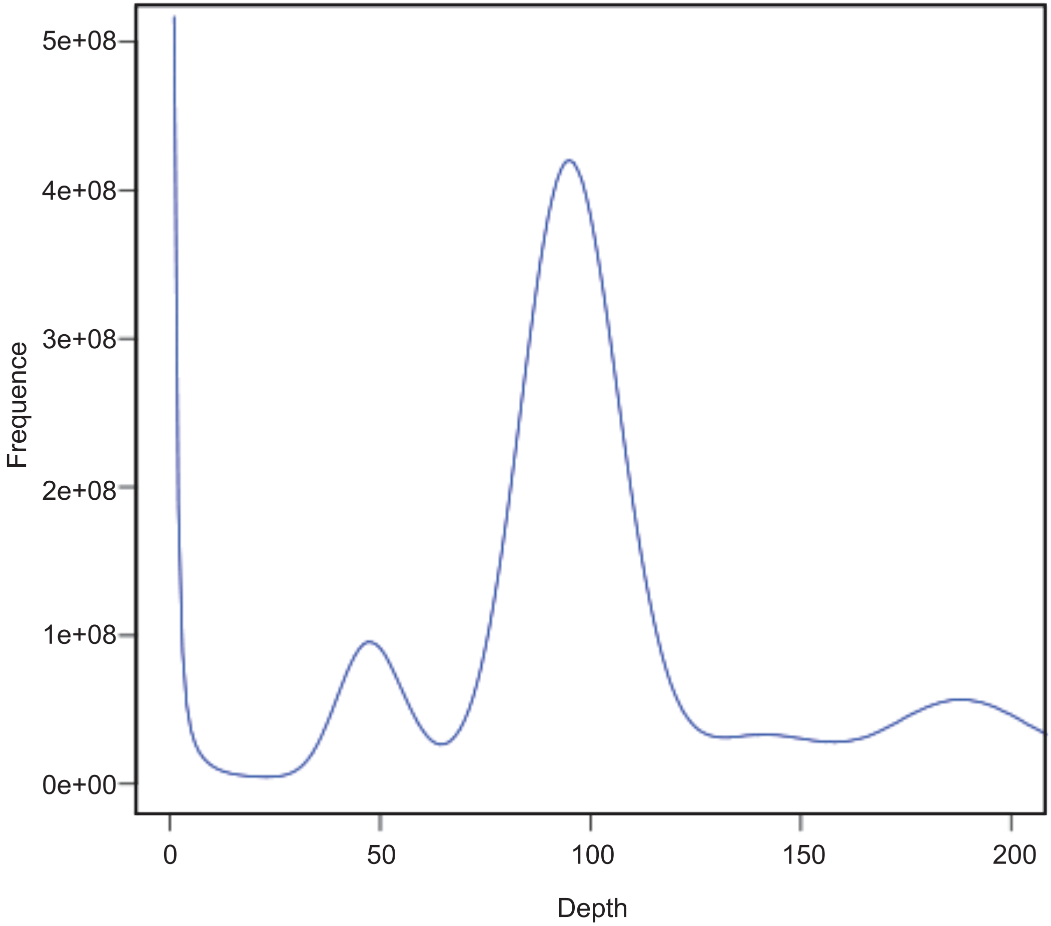

Figure S2. 17-kmer distribution in the lemon genome.

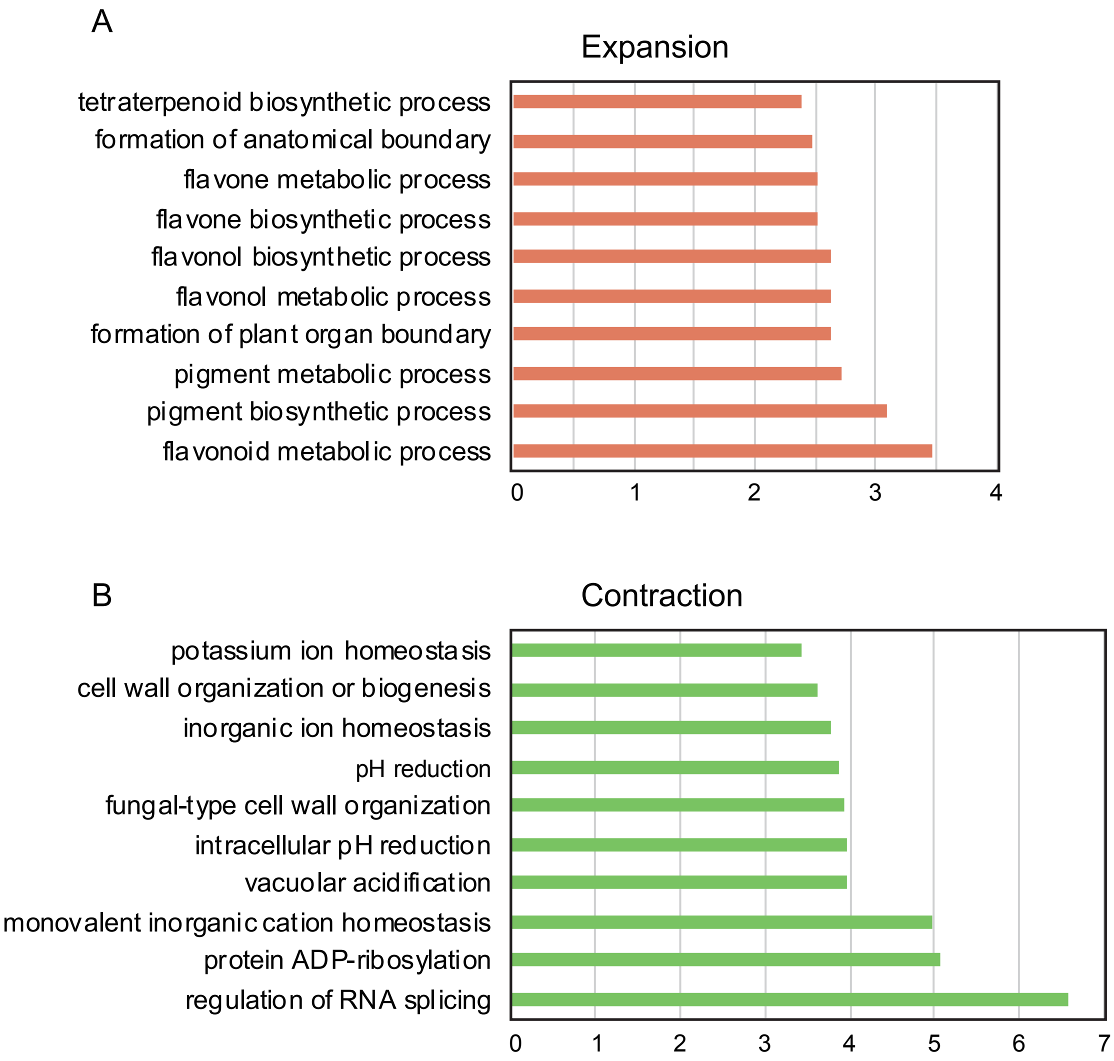

Figure S3. GO enrichment analysis of lemon expansion (A) and contraction (B) gene families.

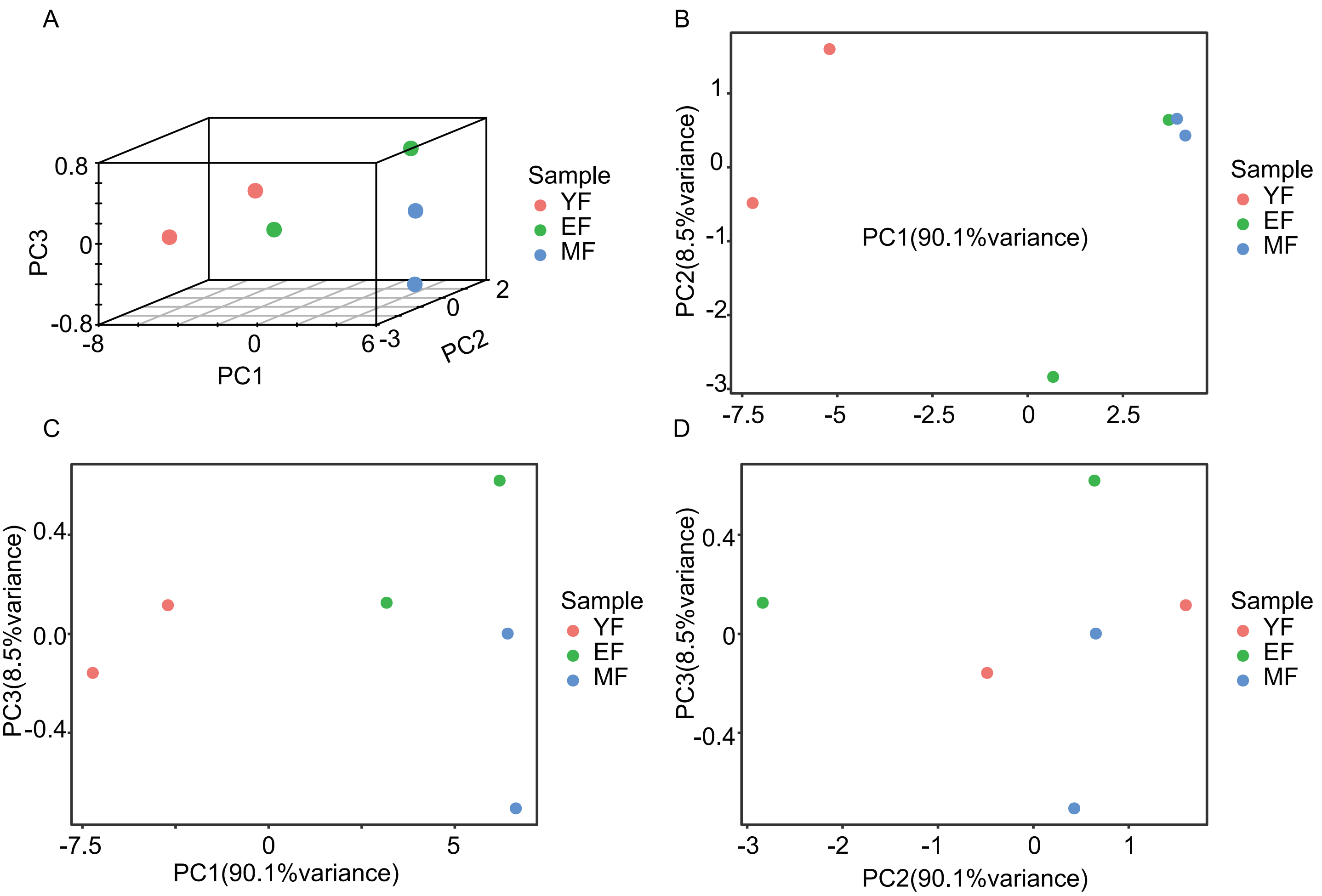

Figure S4. Principal component analysis of all replicates of DNA methylome in YF, EF, and MF.

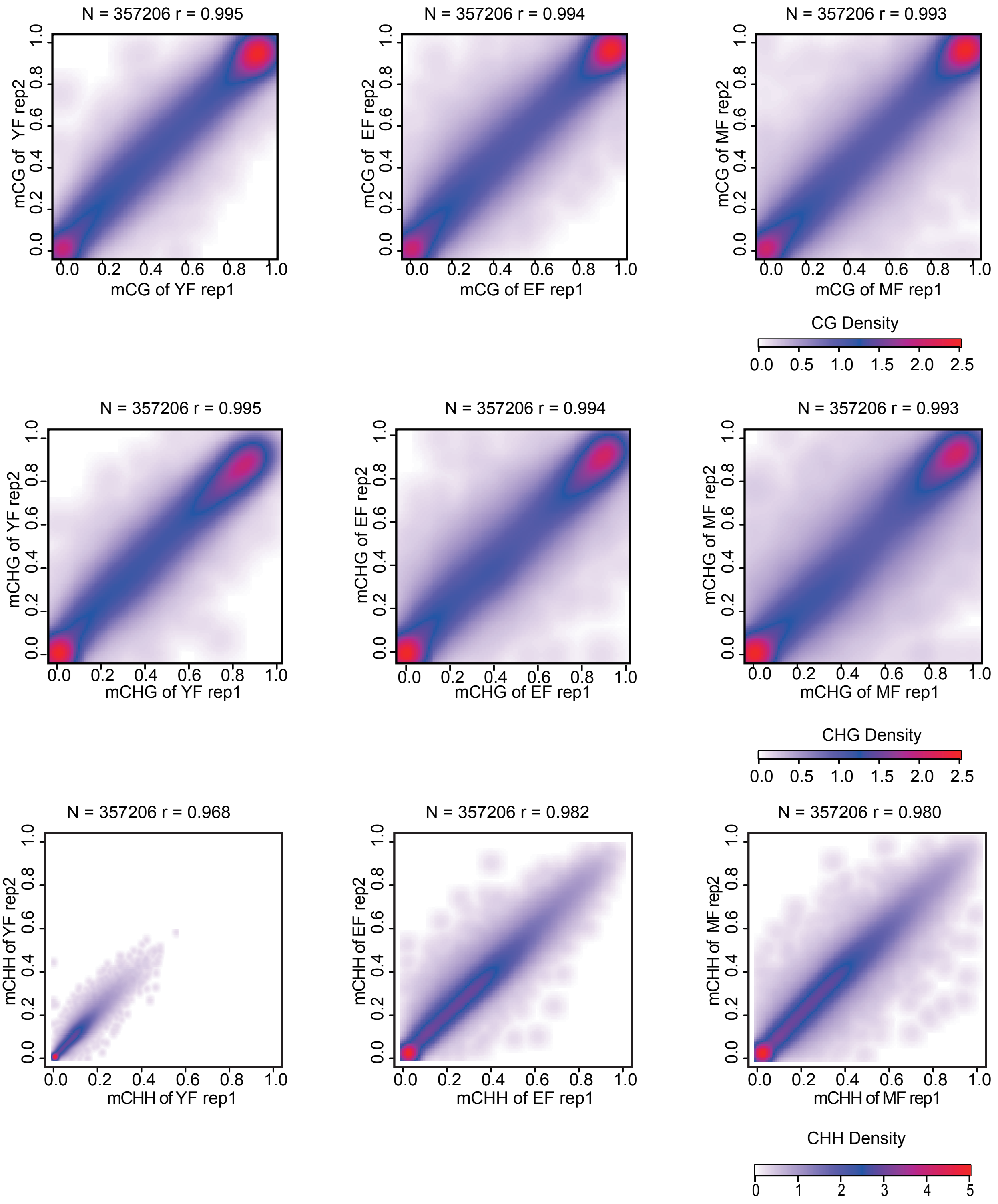

Figure S5. The correlation of replication of BS-seq in lemon.

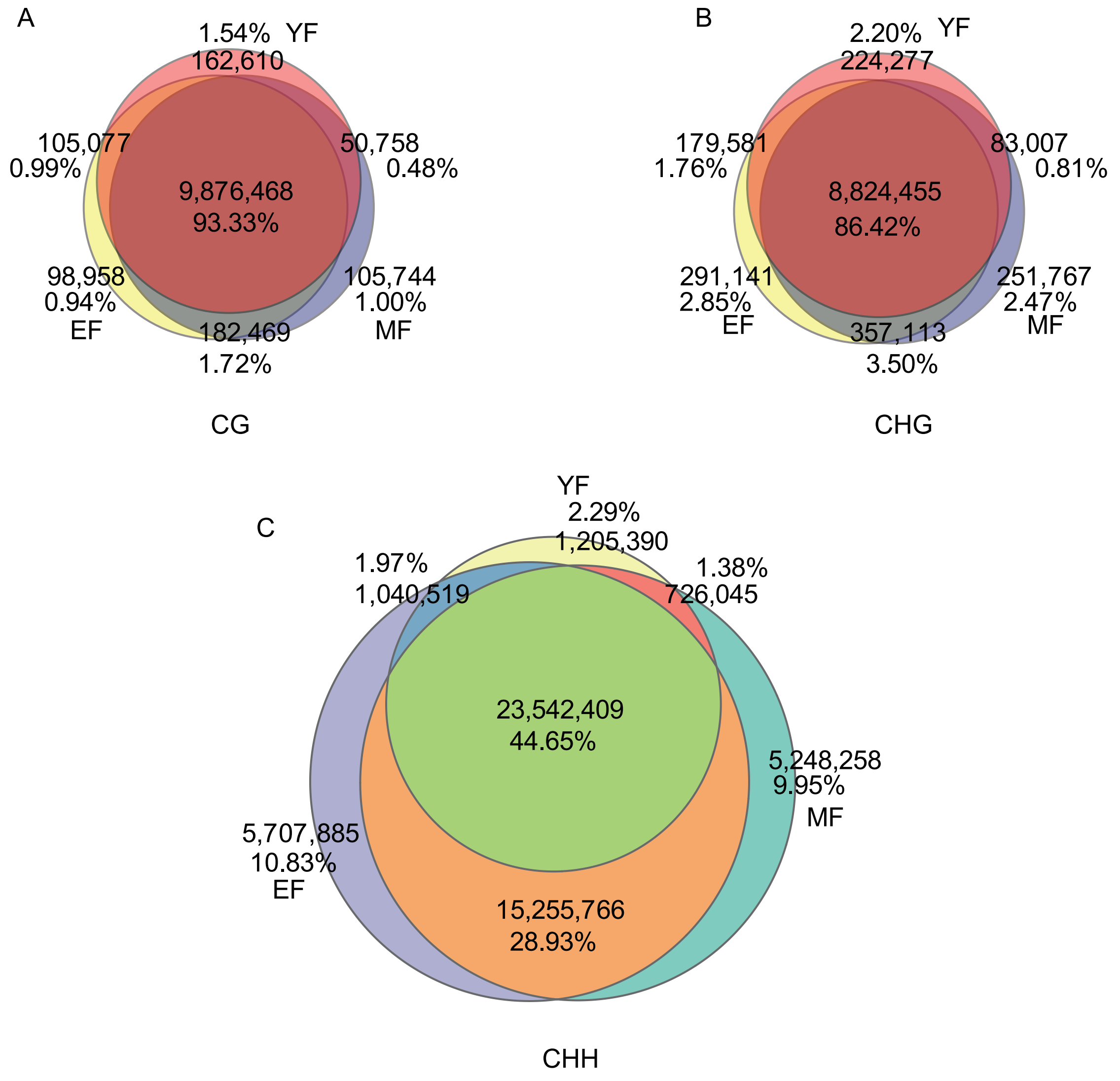

Figure S6. The venn diagrams showing the number of methylcytosine in three tissues of the lemons in CG (A), CHG(B), and CHH(C) contexts.

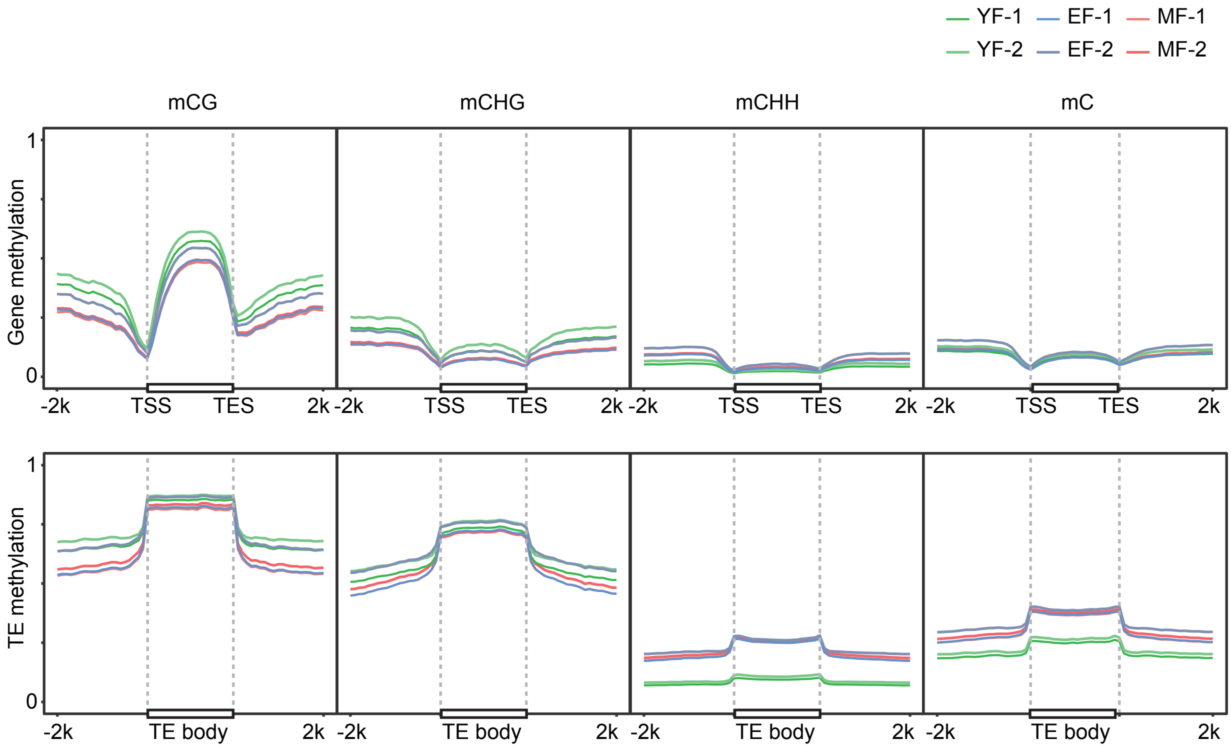

Figure S7. The metaplot showing DNA methylation patterns and levels of protein-coding genes and transposons of each replicate for three different stages.

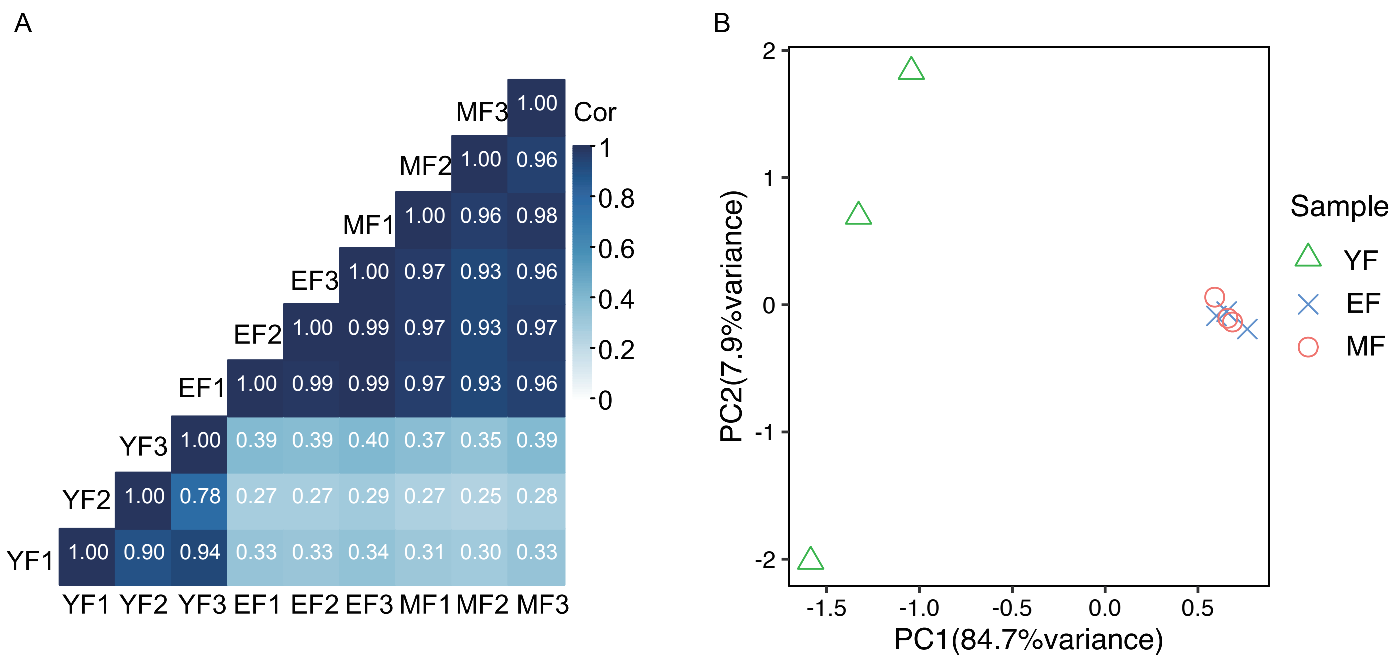

Figure S8. Transcriptome correlation analysis of lemon. (A) Pearson correlation coefficient among three biological replicates of young fruit (YF), expanding fruit (EF), and mature fruit (MF), respectively. (B) Principal component analysis of all nine libraries of transcriptome in YF, EF, and MF.

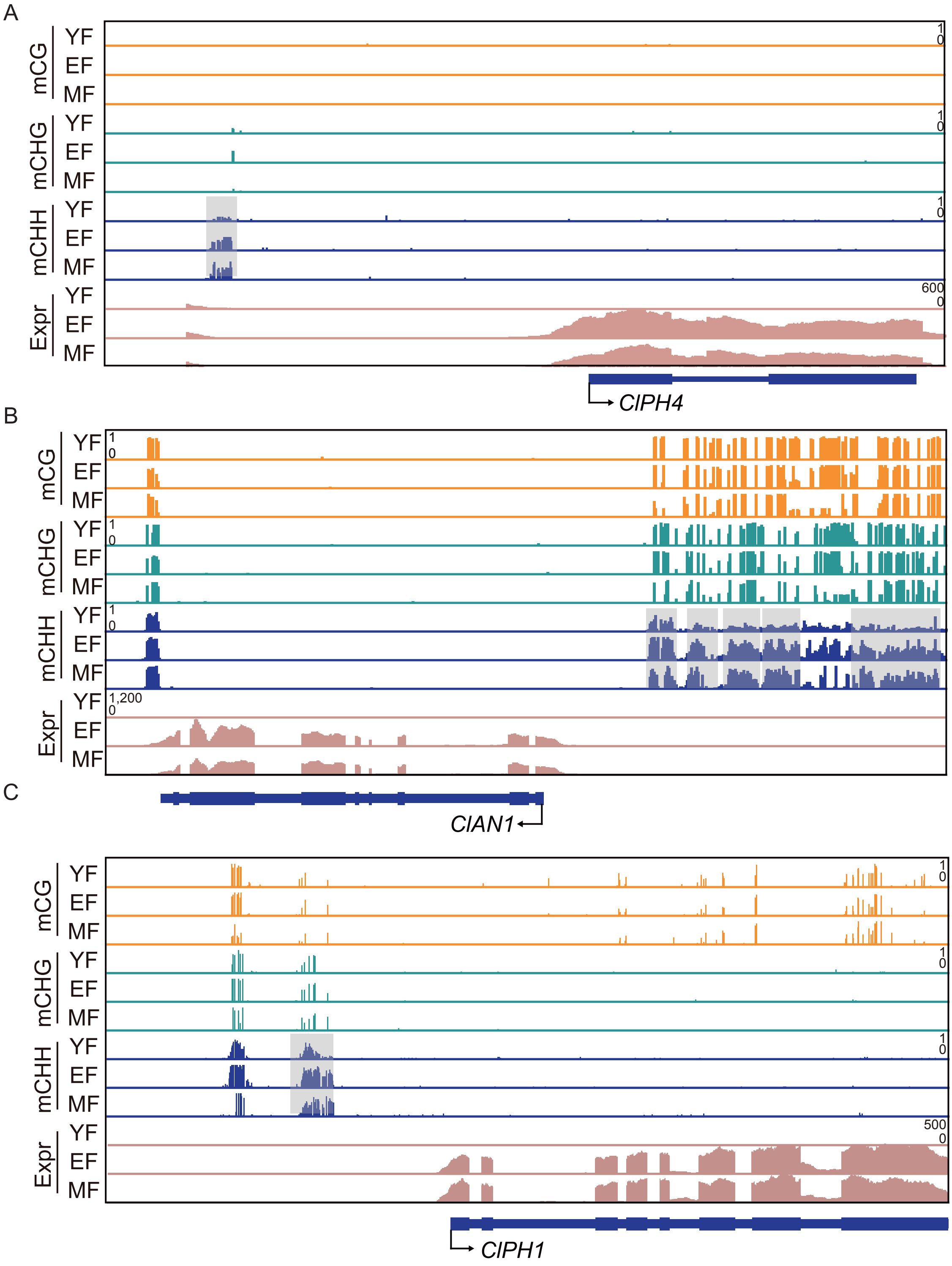

Figure S9. Genome browser showing DNA methylation level and expression level of *CIPH4* (A), *CIAN1* (B), and *CIPH1* (C).
