# Supplementary material for: The lemon genome and DNA methylome unveil epigenetic regulation of citric acid biosynthesis during fruit development
Source: Hortic Res. 2024 Jan 5;11(3):uhae005. doi: 10.1093/hr/uhae005 (PMC10923643; doi:10.1093/hr/uhae005)
Supplement: Web_Material_uhae005 [file web_material_uhae005.zip › Supplementary Table 1-9.docx]

**Supplementary Table 1. Summary of genome assembly.**

| **Sample ID** | **Length** | | **Number** | |
| --- | --- | --- | --- | --- |
|  | Contig (bp) | Scaffold (bp) | Contig | Scaffold |
| Total | 364,849,481 | 364,849,481 | 218 | 218 |
| Max | 17,524,123 | 17,524,123 | - | - |
| Number≥2000 | - | - | 218 | 218 |
| N50 | 3,745,539 | 3,745,539 | 27 | 27 |
| N60 | 2,678,455 | 2,678,455 | 39 | 39 |
| N70 | 2,088,283 | 2,088,283 | 55 | 55 |
| N80 | 1,435,730 | 1,435,730 | 77 | 77 |
| N90 | 905,951 | 905,951 | 109 | 109 |

**Supplementary Table 2. Statistics of clustering number and length of nine chromosomes.**

| **Sequences ID** | **Cluster Number** | **Sequences Length** |
| --- | --- | --- |
| Chr1 | 37 | 53,598,633 |
| Chr2 | 13 | 43,656,661 |
| Chr3 | 20 | 40,612,376 |
| Chr4 | 15 | 39,313,176 |
| Chr5 | 17 | 38,658,008 |
| Chr6 | 17 | 37,083,843 |
| Chr7 | 18 | 35,378,249 |
| Chr8 | 17 | 31,936,480 |
| Chr9 | 18 | 36,972,826 |

*Note: contigs above 100bp is selected to count the assembly results.

**Supplementary Table 3. BUSCO notation assessment results.**

| **Species** | **BUSCO notation assessment results** |
| --- | --- |
| Lemon | C:95.0% [S:85.5%, D:9.5%], F:1.3%, M:3.7%, n:1440 |

Note: C：Complete BUSCOs

S：Complete and single-copy BUSCOs

D：Complete Duplicated BUSCOs

F：Fragmented BUSCOs

M：Missing BUSCOs

n：Total BUSCO groups searched

**Supplementary Table 4. Evaluation of the lemon genome with CEGMA.**

| **Species** | **Complete** | | **Complete+Partial** | |
| --- | --- | --- | --- | --- |
|  | # Prots | %Completeness | # Prots | %Completeness |
| Lemon | 229 | 92.34 | 241 | 97.18 |

  Note: Complete: core gene > 70% assembly;

Complete + partial: partial assembly of core gene;

# Prots: Number of core gene assembled;

%Completeness: Proportion of assembled core gene to core gene library.

**Supplementary Table 5. Statistics of the coverage of Illumina reads in the lemon genome.**

|  |  | **Percentage (%)** |
| --- | --- | --- |
| Reads | Mapping rate (%) | 99.06 |
| Genome | Average sequencing depth | 115.37 |
|  | Coverage (%) | 99.96 |
|  | Coverage at least 4X (%) | 99.92 |
|  | Coverage at least 10X (%) | 99.86 |
|  | Coverage at least 20X (%) | 99.71 |

Note: Mapping rate: the proportion of reads compared to the genome;

Average sequence depth: the average depth of each base on the genome covered by reads;

Coverage: the proportion of the genome covered by reads;

Coverage at least NX (%): the proportion of the genome covered by NX reads.

**Supplementary Table 6. Statistical results of TEs classification.**

| **Type** |  | **Length (bp)** | **Number** | **In Genome (%)** |
| --- | --- | --- | --- | --- |
| DNA |  | 16,592,393 | 42,538 | 4.55 |
| LINE |  | 7,518,424 | 17,338 | 2.06 |
| SINE |  | 51,946 | 147 | 0.01 |
| LTR | Copia | 48,888,569 | 103,973 | 13.40 |
|  | Gypsy | 64,903,645 | 104,374 | 17.79 |
|  | other | 29,394,566 | 43,978 | 8.06 |
| Unknown |  | 11,984,199 | 19,696 | 3.28 |
| Other |  | 3,911,595 | 4,948 | 1.07 |
| Total |  | 176,214,827 | 336,992 | 48.30 |

**Supplementary Table 7. GO enrichment analysis of lemon specific gene families (Top 20 terms).**

| **GO** | **NS** | **Name** | **P value** | **Count** | **FDR** |
| --- | --- | --- | --- | --- | --- |
| 0034237 | MF | protein kinase A regulatory subunit binding | 1.9122E-07 | 4 | 2.44E-04 |
| 0051018 | MF | protein kinase A binding | 1.9122E-07 | 4 | 2.44E-04 |
| 0032760 | BP | positive regulation of tumor necrosis factor production | 2.4751E-06 | 3 | 4.66E-03 |
| 1903555 | BP | regulation of tumor necrosis factor superfamily cytokine production | 2.4751E-06 | 3 | 4.66E-03 |
| 1903557 | BP | positive regulation of tumor necrosis factor superfamily cytokine production | 2.4751E-06 | 3 | 4.66E-03 |
| 0032680 | BP | regulation of tumor necrosis factor production | 2.4751E-06 | 3 | 4.66E-03 |
| 0005879 | CC | axonemal microtubule | 2.4751E-06 | 3 | 1.43E-03 |
| 0032280 | CC | symmetric synapse | 2.4751E-06 | 3 | 1.43E-03 |
| 0050811 | MF | GABA receptor binding | 2.4751E-06 | 3 | 2.11E-03 |
| 0001881 | BP | receptor recycling | 4.9271E-06 | 3 | 7.42E-03 |
| 0098984 | CC | neuron to neuron synapse | 4.9271E-06 | 3 | 1.43E-03 |
| 0032279 | CC | asymmetric synapse | 4.9271E-06 | 3 | 1.43E-03 |
| 0005930 | CC | axoneme | 8.5822E-06 | 3 | 1.66E-03 |
| 0097014 | CC | ciliary plasm | 8.5822E-06 | 3 | 1.66E-03 |
| 0043112 | BP | receptor metabolic process | 1.3667E-05 | 3 | 1.61E-02 |
| 0032838 | CC | plasma membrane bounded cell projection cytoplasm | 1.3667E-05 | 3 | 2.27E-03 |
| 0071490 | BP | cellular response to far red light | 1.4971E-05 | 4 | 1.61E-02 |
| 0043001 | BP | Golgi to plasma membrane protein transport | 2.0406E-05 | 3 | 1.71E-02 |
| 0061951 | BP | establishment of protein localization to plasma membrane | 2.0406E-05 | 3 | 1.71E-02 |
| 0032588 | CC | trans-Golgi network membrane | 2.4363E-05 | 4 | 3.38E-03 |

**Supplementary Table 8. Summary of BS-seq reads mapping.**

| **Sample** | **Total reads** | **Total mapped ratio** **(%)** | **Unique ratio (%)** | **Conversion rate (%)** | **Coverage (X)** |
| --- | --- | --- | --- | --- | --- |
| YF-rep1 | 127,085,876 | 85.94 | 78.14 | 99.41 | 52.24 |
| YF-rep2 | 137,694,786 | 85.89 | 78.59 | 99.42 | 56.60 |
| EF-rep1 | 137,720,440 | 79.51 | 71.41 | 99.72 | 56.61 |
| EF-rep2 | 137,089,430 | 79.90 | 72.90 | 99.70 | 56.35 |
| MF-rep1 | 99,508,398 | 78.99 | 70.79 | 99.71 | 40.90 |
| MF-rep2 | 138,466,246 | 79.30 | 71.40 | 99.71 | 56.92 |

|  | **Total pairs** | **Unique mapped ratio (%)** | **Total mapped ratio (%)** | **Q20 (%)** | **Q30 (%)** | **QC (%)** |
| --- | --- | --- | --- | --- | --- | --- |
| YF-rep1 | 21,442,157 | 85.15 | 95.64 | 98.34 | 94.63 | 44 |
| YF-rep2 | 20,524,982 | 85.34 | 95.07 | 98.51 | 95.09 | 43 |
| YF-rep3 | 24,159,564 | 85.14 | 95.68 | 98.52 | 95.11 | 44 |
| EF-rep1 | 22,153,847 | 87.01 | 96.47 | 98.48 | 94.94 | 45 |
| EF-rep2 | 21,899,903 | 87.04 | 95.99 | 98.49 | 94.98 | 45 |
| EF-rep3 | 19,469,546 | 87.09 | 95.97 | 98.49 | 94.98 | 44 |
| MF-rep1 | 20,155,859 | 86.42 | 95.58 | 98.47 | 94.9 | 45 |
| MF-rep2 | 20,893,533 | 87.39 | 95.97 | 98.28 | 94.46 | 44 |
| MF-rep3 | 24,558,660 | 87.28 | 95.95 | 98.32 | 94.58 | 44 |

**Supplementary Table 9. Summary of RNA-seq reads mapping.**
